# Apple Leave Disease Detection Using Collaborative ML/DL and Artificial Intelligence Methods: Scientometric Analysis

**DOI:** 10.3390/ijerph20043222

**Published:** 2023-02-12

**Authors:** Anupam Bonkra, Pramod Kumar Bhatt, Joanna Rosak-Szyrocka, Kamalakanta Muduli, Ladislav Pilař, Amandeep Kaur, Nidhi Chahal, Arun Kumar Rana

**Affiliations:** 1 Amity School of Engineering and Technology, Amity University Rajasthan, Jaipur 303002, India; 2 Chandigarh Engineering College, Chandigarh Group of Colleges, Landran, Mohali 140307, Punjab, India; 3 Department of Production Engineering and Safety, Faculty of Management, Częstochowa University of Technology, 42-200 Częstochowa, Poland; 4 Department of Mechanical Engineering, Papua New Guinea University of Technology, Lae 411, Morobe, Papua New Guinea; 5 Department of Management, Faculty of Economics and Management, Czech University of Life Sciences Prague, 16500 Prague, Czech Republic; 6 Chitkara University Institute of Engineering and Technology, Chitkara University, Rajpura 140417, Punjab, India; 7 Computer Science and Engineering, Galgotias College of Engineering and Technology, Greater Noida 203201, India

**Keywords:** apple leaves disease detection, deep learning, machine learning, bibliometric, scientific mapping, bibliographic coupling, VOSviewer

## Abstract

Infection in apple leaves is typically brought on by unanticipated weather conditions such as rain, hailstorms, draughts, and fog. As a direct consequence of this, the farmers suffer a significant loss of productivity. It is essential to be able to identify apple leaf diseases in advance in order to prevent the occurrence of this disease and minimise losses to productivity caused by it. The research offers a bibliometric analysis of the effectiveness of artificial intelligence in diagnosing diseases affecting apple leaves. The study provides a bibliometric evaluation of apple leaf disease detection using artificial intelligence. Through an analysis of broad current developments, publication and citation structures, ownership and cooperation patterns, bibliographic coupling, productivity patterns, and other characteristics, this scientometric study seeks to discover apple diseases. Nevertheless, numerous exploratory, conceptual, and empirical studies have concentrated on the identification of apple illnesses. However, given that disease detection is not confined to a single field of study, there have been very few attempts to create an extensive science map of transdisciplinary studies. In bibliometric assessments, it is important to take into account the growing amount of research on this subject. The study synthesises knowledge structures to determine the trend in the research topic. A scientometric analysis was performed on a sample of 214 documents in the subject of identifying apple leaf disease using a scientific search technique on the Scopus database for the years 2011–2022. In order to conduct the study, the Bibliometrix suite’s VOSviewer and the web-based Biblioshiny software were also utilised. Important journals, authors, nations, articles, and subjects were chosen using the automated workflow of the software. Furthermore, citation and co-citation checks were performed along with social network analysis. In addition to the intellectual and social organisation of the meadow, this investigation reveals the conceptual structure of the area. It contributes to the body of literature by giving academics and practitioners a strong conceptual framework on which to base their search for solutions and by making perceptive recommendations for potential future research areas.

## 1. Introduction

The growth of the global economy depends heavily on the agricultural sector. The planet is suffering from widespread environmental neglect. Hailstorms, fog, and sudden rain are just a few examples of environmental uncertainty that can completely destroy crops or spread infectious diseases [1]. Plant disease, which is one of the main causes of crop damage and ultimately drives farmers into poverty and suicide, is the biggest and most intricate issue. Crop losses also have a detrimental effect on the world economy [2].

In the past, farmers and agricultural specialists employed physical and visual inspection techniques to spot diseases. These methods had some problems, such as taking a long time and being hard to use. The results could only be used in a small area.

Technology plays a significant role in the agricultural sector, businesses built around it, and the expansion of farms. Growing food even in deserts is now possible thanks to recent agricultural technological breakthroughs. The agricultural sector has the highest demand for automation methods. Numerous studies have shown that the use of computerization technology on farms will boost agricultural output and, consequently, farmers’ yearly revenue [3,4,5].

Agriculture is only one of the many industries where artificial intelligence (AI) is a major force. The majority of common agricultural issues can be solved utilising AI [6]. It helps with the early diagnosis of a variety of leaf diseases. Farmers may increase yields while decreasing losses if they use autonomous apple leaf disease detection equipment. On this issue, several researchers have worked. To identify agricultural illnesses, the majority of them tried different machine learning, image processing, and deep learning techniques [7].

China is the most prolific fruit producer on the globe, and both the whole planted vicinity and the entirety of the apple succumb rank first worldwide [7]. The apple leaf, nonetheless, is simply diseased and infested with parasites and diseases, including ring rot, grime, early dethatching, scab, and others. This has been possible in recent years because of the development of deep learning technology and the advancement of computer data processing capabilities. By combining information such as colour, shape, texture, and other features to create feature vectors and then classifying the feature vectors using an artificial neural network (ANN) [8], a support vector machine (SVM) [9], or other methods, it is possible to achieve a specific classification effect. To automatically identify agricultural diseases, including those that impact rice, corn [9], wheat [10], cotton [11], tomato [11], and cucumber [12], researchers are utilising image processing, machine learning, and other techniques. Numerous feature segmentation techniques, such as k-means clustering [10], fuzzy C-means [11], Roberts detection, Prewitt detection [12], and Sobel detection and extraction techniques [13], such as Tamura, Entropy [14], RMS [15], and Kurtosis [16], are used to detect diseases as a result of technological advancements [17]. These techniques allow farmers to automatically identify the various diseases that affect particular crops.

### 1.1. Apple Leaves Disease Categories

**Figure 1:**
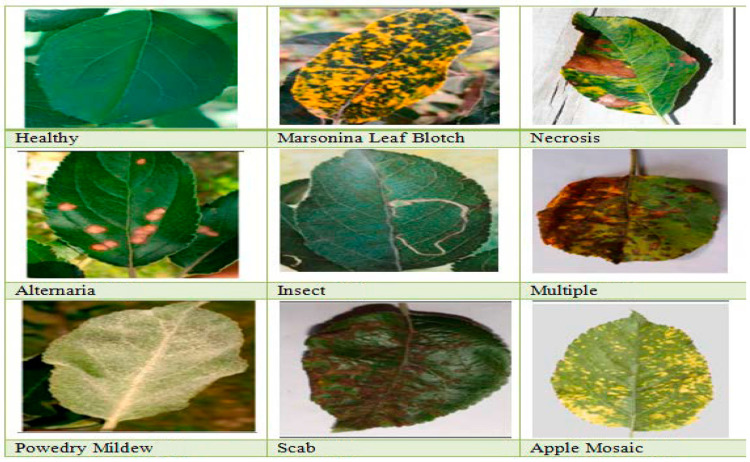
Diseases that Affect Apple Leaves, by Types [18,19,20,21,22,23,24,25].

The various types of apple leaf disease with their diagrammatical representation are described using the phrases below in Figure 1.

#### 1.1.1. Apple Healthy Leaves

Apple leaves that fall into this category are flawless, green, and free of any disease-related defects. According to the researchers’ findings, the ratio of healthy leaves in an apple is approximately 28% [18].

#### 1.1.2. Marsonina Leaf Blotch

A harmful fungus called apple Marsonina blotch (AMB), which results in early defoliation and causes fruit to shrink and develop less starch. Since it was initially identified in Japan, the pathogen that causes AMB, Diplocarpon mali, has been rapidly spreading throughout the world [19].

#### 1.1.3. Apple Necrosis Leaves

It is a physiological state that affects the crop because of an imbalance of soil moisture, light intensity, and air temperature [18].

#### 1.1.4. Apple Alternaria Leaves

It also goes by the names “black rot” and “frogeye leaf spot,” and it is brought on by the Physalospora obtuse virus. The cause of this disease is a fungal infection that affects several stages of crops [20].

#### 1.1.5. Powdery Mildew

When the buds transform into fresh leaves and stems, this type of ailment begins to manifest. There are only a few patches of grey or white chalky aggregates on the underside of the leaves. The leaves become longer and thinner than typical leaves when the border is curled [23].

#### 1.1.6. Apple Scab

The earliest signs of this serious fungal ailment, which is most common in spring and is caused by Venturia inadequacies, are tiny scratches on the bases of the leaves. Before moving on to the fruit, where it appears as microscopic black scabs, it spreads to the tips of the leaves [24].

#### 1.1.7. Apple Mosaic

Apple mosaic is a pathogenic and positive-sense RNA virus. In response to this virus, apple trees often develop light yellow erratic patches or streaks on their leaves in the spring, and it spreads when the weather’s temperature is out of balance [25].

#### 1.1.8. Multiple Diseases

These ailments include scabs, which have little brown dots, and cedar apple rust, which has yellow patches. The foliar disease category includes this kind of infection [22].

**Figure 2:**
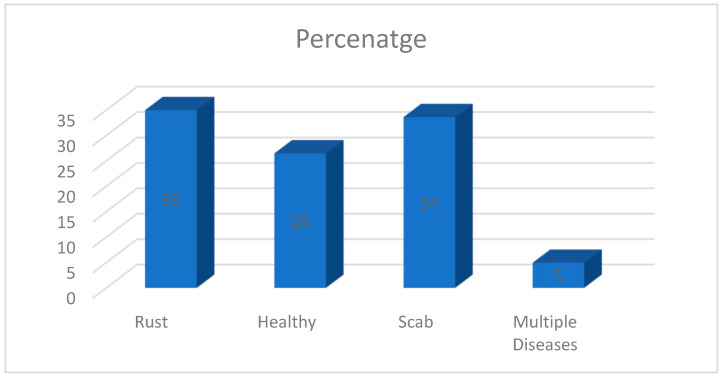
Distribution of classes of Apple leaves disease in Plant Pathology 2020 dataset [26,27].

There are 1821 photos of apple leaves distributed proportionally among four labels or classifications, including rust, healthy, scab, and many diseases, in the Plant Pathology 2020-FGVC7 dataset [26], which is openly accessible via Kaggle. There are 289 images in the healthy class, 382 in the rust class, 367 in the scab class, and 54 in the numerous diseases class out of 1092 total photographs. The distribution of the classes is shown in Figure 2.

The majority of the aforesaid diseases affect apple leaves, and an automatic system will be developed to make it simple to detect them. In comparison to the conventional way, adopting automation devices makes the identification process relatively simple and saves farmers’ time and effort [28].

According to Statista.com, global fruit production volume has steadily increased over the last two decades. Between 2010 and 2020, the global volume of fruit produced increased from 750.5 million metric tons to approximately 887 million metric tons. China is by far the largest producer of fresh fruit. India was the second largest fruit producer, followed by Brazil.

China was the world’s leading apple producer in the crop year 2021/2022. During that time period, China produced nearly 46 million metric tons of apples. With approximately 12.28 million metric tons of apples, the European Union came in second place. “Global top apple producing countries 2022 | Statista.” https://www.statista.com/statistics/279555/global-top-apple-producing-countries/ (accessed on 3 February 2023).

**Figure 3:**
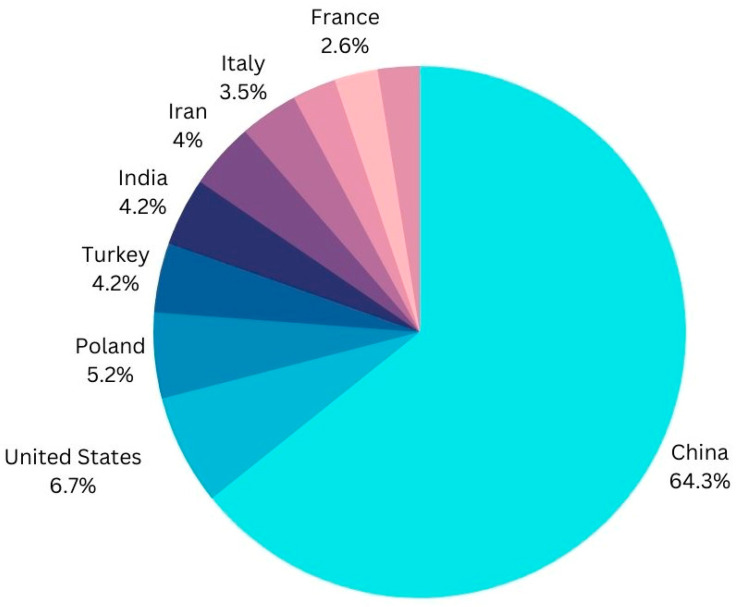
Universal Most and Least production rate of Apple Fruit.

The production rate of apples around the world is depicted in the Figure 3 below.

Therefore, it has become crucial for researchers to focus on illness detection in order to protect the global economy. The given data show that, among all nations, China has the highest rate of production, followed by the United States, Poland, India, Italy, and France. There are numerous strategies used for apple leaf disease detection by using AI features such as ML/DL [29], data preprocessing [30], picture segmentation [31], image classification [32], and feature extraction [33] in order to save crops and sustain the global economy, and all information will be shared and collected by IoT devices collaborating with the cloud so that future work will be time-saving [33,34,35]. Since more than 20 years ago, there has been intense and ongoing research into apple integrated pest management (IPM) and the development of apple IPM programmes through the transfer technology activities of Extension and Advisory personnel and crop consultants [36]. On both pome and stone fruits, Botryosphaeria species produce fruits and canker. Bovine apple canker had a minor impact in the United States until 1952 [37]. To recognise and keep track of economically significant apple illnesses, short DNA gene sequences (oligonucleotides) from the ribosomal spacer sections of bacterial and fungal pathogens were employed [38]. In semi-commercial and commercial experiments, the yeasts Rhodotorulaglutinis (strain HRB6), Cryptococcus laurentii (strain HRA5), and Cryptococcus infirmominiatus (strain YY6) were examined as potential biocontrol agents for postharvest diseases of apples and pears [39].

The study followed by brief justification about the deep learning techniques, image segmentation and how they will implement for detection of disease in sophisticated manner.

### 1.2. Deep Learning Techniques

In supervised learning, unsupervised learning, reinforcement learning, and hybrid learning, deep neural networks perform well.

In supervised learning, the mapping function f is learned using an algorithm, and the input variables represented as X are mapped to the output variables represented as Y.

Y = f(X)(1)

To forecast the output (Y) given a new input, the learning algorithm aims to approximate the mapping function (X). The output can be corrected using the error from the training-related predictions. Learning can be halted once all inputs have been trained to produce the desired output [40]. Regression [41] is used to solve regression problems; Support Vector Machines [42] is used to solve classification difficulties; and Random Forest [43] is used to solve both classification and regression problems.

With unsupervised learning, there are no corresponding outputs to map; only the input data are available. The goal of this learning is to gain knowledge of data by simulating its distribution. The fascinating structure that exists in the data can be found using algorithms. Unsupervised learning is used to solve clustering and association problems. Unsupervised learning methods such as the K-means algorithm, the Apriori algorithm, and the Random Forest algorithm are used in clustering problems [44], association problems [45], classification problems [42], and regression problems [43].

The algorithm is trained via reinforcement learning, which employs a reward and punishment scheme. The algorithm or agent in this situation picks up information from its surroundings. For good performance, the agent receives bonuses; for bad performance, they receive penalties. Consider the situation of a self-driving automobile, where the agent receives a reward for reaching the destination safely and a penalty for driving off the road. Similar to the last example, chess software might have a reward state of winning and a penalty state of being checkmated. The agent aims to reduce the punishment and increase the reward. The algorithm learns on its own through reinforcement learning without being instructed on how to proceed [46].

Architectures that use both discriminative (supervised) and generative (unsupervised) components are referred to as “hybrid learning architectures.” A hybrid deep neural network can be created by combining various architectures. They are expected to provide considerably better results [47] when used to recognise human actions using action bank features. They are instructed on how to carry out the learning, but it solves the issue on its own [46].

### 1.3. Image Segmentation

A pre-processing method used in digital image processing is called image segmentation [48]. It is a technique that divides an image into several interest zones based on specific traits for example, vigour, colour, or texture.

**Figure 4:**
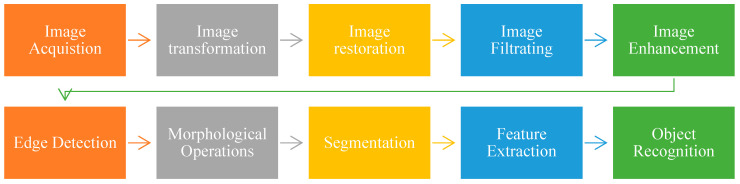
Various Levels of Digital Image Processing [49].

By dividing an image into segments, it is possible to distinguish these segments from the background [49]. Figure 4 illustrates basic steps for segmenting images into different segments and helps to obtain quality image for further work. There are numerous techniques are followed for segmenting images, some of are given below.

#### 1.3.1. Edge Detection Segmentation

**Figure 5:**
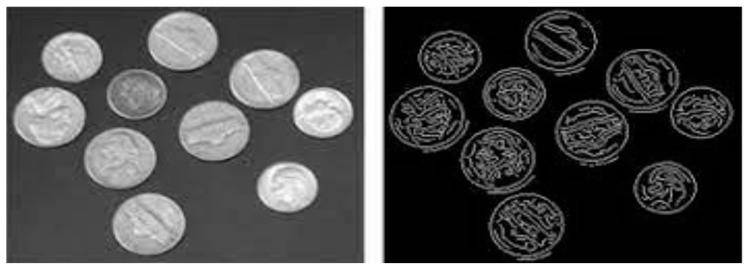
Edge Detection Segmentation [50].

An initial step in the image segmentation process is edge detection. It delineates the boundary between an image’s subject and background. Edge detection splits an image by identifying changes in brightness or pixel count shown in a given Figure 5 [50].

#### 1.3.2. Thresholding Segmentation

**Figure 6:**
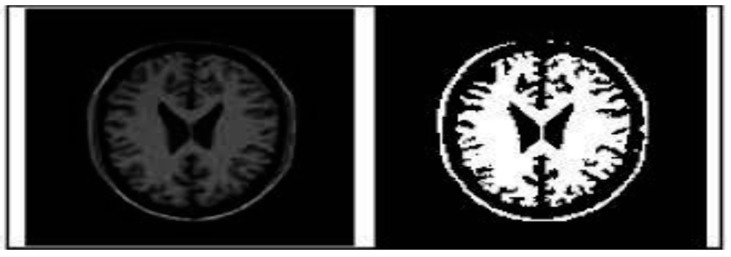
Thresholding Segmentation [50].

Based on intensity levels, a method known as threshold-based segmentation is one of the simplest ways to divide up an image [50]. The below Figure 6 will illustrate this segmentation.

#### 1.3.3. Region-Based Segmentation

**Figure 7:**
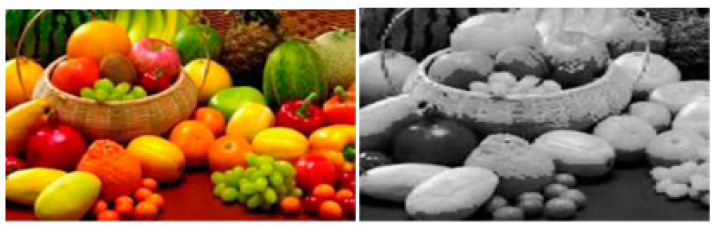
Region-Based Segmentation [50].

This approach groups pixels that are associated with the same item to segment data. The thresholding method is related to the region-based segmentation approach. The region that was discovered for segmentation should be sealed off [50]. The below Figure 7 will illustrate this segmentation.

#### 1.3.4. Feature-Based Clustering Segmentation

**Figure 8:**
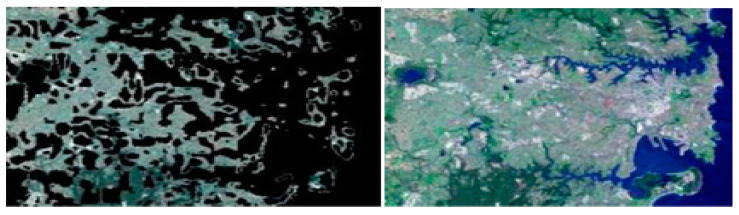
Feature-Based Clustering Segmentation [50].

The approach of organising the groupings according to their characteristics is called clustering. A cluster is frequently made up of a collection of related pixels that are distinct from pixels in other locations and are concentrated in one place which is shown in below Figure 8 [50].

### 1.4. Need of Bibliometric Study

A common and reliable method for reading and analysing vast amounts of the scientific literature is bibliometric analysis. The development, accessibility, and availability of bibliometric tools such as Gephi, Leximancer, Biblioshiney, and VOSviewer, as well as scholarly databases such as Scopus and Web of Science, have all contributed to the recent explosive growth in popularity of bibliometric analysis [51].

This is also a product of the interdisciplinary cross-pollination between business research and information science that occurs in the bibliometric scientific approach. Researchers utilise bibliometric analysis for a variety of purposes, including studying the theoretical underpinnings of an existing field and recognising new trends in the performance of publications and journals, collaboration patterns, and research constituents [52].

### 1.5. Bibliometric Analysis Apple Leaves Disease Detection using Artificial Intelligence

In view of the aforementioned issue, the researchers feel forced to execute a bibliometric study and comprehend the research allied to the detection of plant diseases with artificial intelligence. It is customary to list the sources at the end of a book, article, or report to demonstrate that the data from those sources were used to construct that particular book, article, or report [53]. The words “biblio” and “metrics,” which stand for assessment and the literature, respectively, make up the term “bibliometrics.” It is employed to assess how well institutions’ and organisations’ research is performing. It is a frequently employed technique for precisely locating research data depending on the quantity of articles, the quantity of citations, the locations of the critiques, and several other features [51]. This study also assists in identifying areas for future research and possible directions for the authors’ contributions to the subject.

The following are the major goals of this work:

- To identify the many research publishing categories.
- To determine the linguistic style used in publications.
- To identify publication trends based on year.
- To pinpoint the regions or nations that has contributed more to the study.
- To identify patterns using various source kinds.
- To identify authors who contribute significantly.
- To spot publishing trends based on connections (college/organisation).
- To look at the publication’s citation counts.

In Chapter 2, which focuses on the early data collection on the disease, this article provides a bibliometric review of apple leaf diseases. The investigation of the salvages Scopus data is the main topic of Chapter 3. Network analysis and statistical analysis are the two basic types of analyses performed in this area. The research’s results are discussed in Chapter 4. The survey’s boundaries are illustrated in Chapter 5, and the study paper’s conclusion is depicted in Section 6 the bibliography at the conclusion of the essay.

## 2. Research Methodology

**Figure 9:**
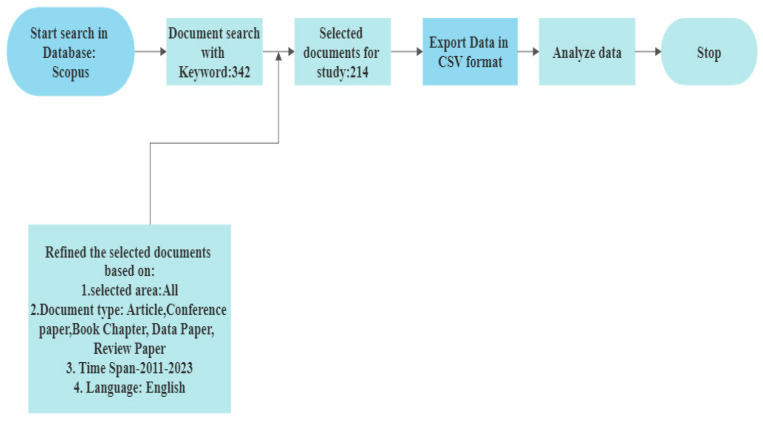
Procedure for Selection of Documents from Scopus Database.

The study will proceed through data gathering and selection. To design the search strategy for completing this work, use the flow chart below in Figure 9.

### 2.1. Initial Data Collection

A database is a planned collection of data. It falls within the multidisciplinary and specialised categories. Between 2011 and 2022, Elsevier’s extensive Scopus database acted as the study’s source of data (through September, October) [54]. There are two ways to access scholarly publications such as articles and documents. The necessary object can either be accessed for free (often known as “open access”) or by paying a fee to access it. Researchers can gain access to the publications by signing up on the appropriate websites or by visiting the library portals of the organisations or institutes. There appear to be a couple additional ways to obtain the information from research databases. Scopus, Web of Science, Science Direct, Research Gate, and Google Scholar are a few of the well-known research databases.

One of the biggest collections of scientific publications, including journal articles, conference materials, and book chapters, is Scopus. Peer-reviewed articles come from a variety of academic disciplines, including science, medicine, the arts, and engineering, and are evaluated by subject-matter experts. The entire world is covered by it. Scopus is regarded as a trustworthy and dependable source whenever research is required as a result. Over 3000 academic, governmental, and commercial entities use Scopus, which serves as the main data source for the Elsevier Research Intelligence portfolio [54]. By connecting the significant keywords listed in Section 2.2 to the Scopus database, the analysis takes the database into consideration.

### 2.2. Significant Keywords

**Figure 10:**
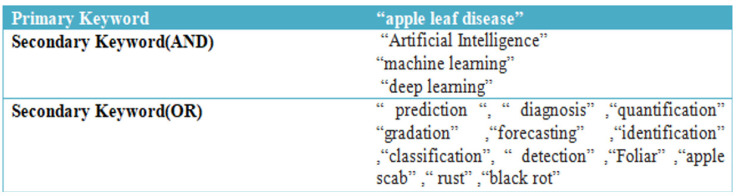
Primary and Secondary Search Strings.

The primary and secondary terms were used to categorise the common terminologies used to describe apple leaves diseases. The search phrases used for this study’s methodology are listed in given Figure 10.

Thus, the following search term is used to look up documents in Scopus: “apple leaf disease” AND “Artificial Intelligence” OR “machine learning” OR “ deep learning” OR “ prediction” OR “diagnosis” OR “quantification” OR “gradation” OR “forecasting” OR “identification” OR “classification” OR “detection” OR “foliar” OR “apple scab” OR “rust” OR “black rot”.

**Figure 11:**
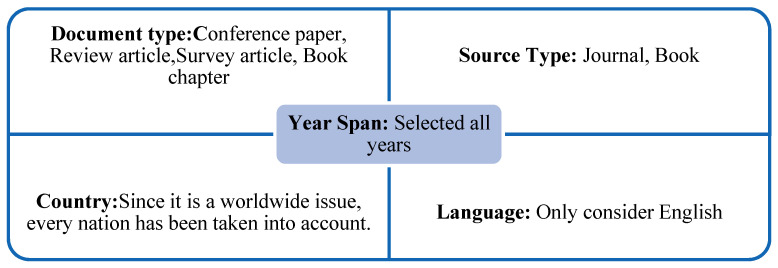
Basic Principles of Data Collection.

There are certain fundamental standards to adhere to when searching the data in the Figure 11 below:

Some common analysis tools, some of which are explained in the next phrase, are relevant for the efficient conduct of this investigation.

### 2.3. Bibliometric Analysis Tools

Since there are numerous bibliometric review tools, each one is distinctive in its own manner. As we all know, plain text is less appealing than visual representation. Understanding the relationships between study components was made easier by using bibliometric analysis with graphical representation or scientific map representation [55]. Even though the research can be conducted with basic social network analysis software, there are different software solutions built expressly for science mapping. Several programmes include the following.

#### 2.3.1. Bibexcel

At the University of Ume, Bibexcel, a bibliometric tool, was established (Sweden). This tool was created with the express purpose of managing bibliometric statistics and producing plots that can be examined by programmes such as Excel [56], SPSS [57], UCINET [58], and Pajek [59]. In educational settings, Bibexcel can be utilised without charge. A number of bibliographic sources, such as ISI Web of Science (WoS), Scopus, and the Procite export format, can be read by Bibexcel.

The textual data can be subjected to a number of preparation activities with the help of Bibexcel, including the use of an English word stemmer and the elimination of redundant documents. Additionally, Bibexcel allows for the periodic removal of components while only keeping the strongest connections [60].

#### 2.3.2. CopalRed

The scientific community EC3 at the University of Granada created the commercial programme Copal Red (Spain) [61]. The co-word analysis it performs utilising the keywords in scientific texts is its intended use. It is referred to as a learning system that gathers data from databases and turns it into fresh knowledge.

This software programme can read Comma-Separated Values (CSV) files [62] produced by the benchmark supervisor program. One perk of Copal Red is that it features a preprocessing module that makes it simple for the user to normalise the keywords.

#### 2.3.3. CiteSpace

CiteSpace is a free download that was created at Drexel University in the United States [63,64]. It is freeware designed to find, examine, and display trends and patterns in the research literature. Its major objective is to make it easier to analyse new trends in a field of expertise. Several citation formats, including WoS, PubMed, arXiv, and the SAO/NASA Astrophysics Data System, can be read by CiteSpace (ADS). Among other sources, CiteSpace can extract bestow data from NSF Awards and Derwent Innovations Index patent data.

#### 2.3.4. IN-SPIRE

IN-SPIRE is a for-profit aesthetic archival research tool that enables analysts to find connections, patterns, and themes in data to gain new understanding and fresh perspectives [65]. In order to make it simple for the user to understand the relationships between documents and groups of documents that are quite similar, IN-SPIRE makes use of the idea of a landscape. This programme classifies articles according to their context using statistical word patterns. IN-SPIRE can read both ASCII text and documents with formatting, such as HTML [66] and XML [67]. Additionally, it can actually read data from CSV-structured files and Microsoft Excel spreadsheets [68].

#### 2.3.5. Sci2 Tool

A collection of modular tools called the Sci2 Tool was developed specifically for conducting scientific research. It includes network analysis in addition to chronological, geographic, thematic, and local analysis and allows the presentation of records at the femto (individual), meso (local), and mega (global) levels. It was created by Indiana University’s Network Science Center’s cyber infrastructure and is publicly available [69]. Using a variety of plug-ins and layout techniques, the Sci2 Tool enables data pretreatment and preparation, network extraction, temporal, geographic, topical, and network analysis, as well as the visualisation of the outcomes. The DRL layout methodology is present in the Sci2 tool.

#### 2.3.6. Bibliometrix

A special programme called Bibliometrix was created in the R statistical computing and graphics language [70] in accordance with a logical bibliometric methodology. Implement bibliometric analysis and create data matrices for co-citation, coupling, scientific collaboration analysis, and co-word analysis, among other activities, using the Bibliometrix software. Bibliographic information may be imported from the Scopus [71], PubMed [72], Digital Science Dimensions, Web of Science, and Cochrane databases [73], among others.

Even individuals without coding experience can utilise bibliometrics owing to the shiny web app Biblioshiny. “Bibliometrix for no coders” is Biblioshiny. It makes it simple for academics to utilise Bibliometrix’s key capabilities, including data importation, analytics, and graphs for triple cognitive structures (K-structures): logical structure, cerebral structure, and class fabric, using data files from WoS, Scopus, PubMed, Lens, and dimensions [74].

#### 2.3.7. VOSviewer

VOSviewer is a piece of software created especially for creating and displaying bibliometric maps, with a focus on their graphical depiction. Considering the usage of density metaphors, special labelling algorithms, and zoom functionality, huge maps can be represented appropriately. The bibliometric research community can use the software application for free. Leide University’s Center for Science and Technology Studies was responsible for its creation (the Netherlands). The things are organised on the maps using VOSviewer’s VOS mapping technology. The method generates an identical matrix from a co-occurrence matrix using the association strength similarity measure [75].

The tools Biblioshiny and VOSviewer are utilised in the execution of this study to obtain the best outcomes.

## 3. Bibliometric Analysis and Results

**Figure 12:**
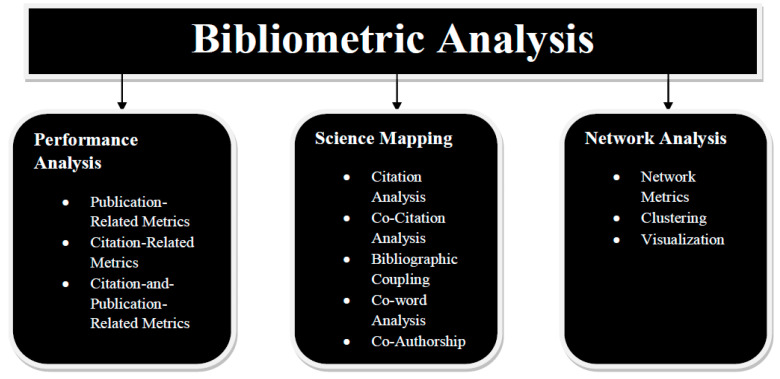
Various forms of bibliometric analysis.

The approaches for bibliometric investigation fall into two groups: performance analysis and science mapping. Science mapping places more emphasis on the connections between research aspects than performance analysis, which primarily examines the contributions of research components [76]. The approaches for analysis methods and scientific mapping, which are illustrated in Figure 12.

### 3.1. Performance Analysis

The contributions made by several study participants in a certain field are evaluated through performance research. Descriptive analysis is the cornerstone of bibliometric studies. Reviews typically discuss how various study participants performed, including authors, institutions, countries, and specialised journals. This is similar to the milieu information or participant silhouette that is characteristically provided in pragmatic research but is presented in a more analytical manner. As a result, even non-science mapping evaluations frequently include performance analysis [77].

Multiple performance analysis measures are offered. Publications and referrals, either annually or per research component, are the most popular metrics. Publications serve as proxies for productivity, and citations serve as indicators of impact and influence. In order to evaluate the effectiveness of research contributors, two additional metrics that combine citations and publications are the h-index and the number of citations per article [78].

### 3.2. Science Mapping

The links between many scientific fields are examined by science mapping [79]. The main focus of the analyses is on the structural relationships and intellectual exchanges between the research component parts.

Among the techniques employed in scientific mapping are citation scrutiny, co-citation investigation, bibliographic coupling [80], co-word analysis, and co-authorship analysis. When these techniques are used with network analysis, the bibliometric organisation and intellectual structure of the research topic can be more clearly understood.

#### 3.2.1. Citation Analysis

Citation analysis [81], which is based on the idea that citations signify the intellectual links between publications as a result of one article referring to another, is a crucial technique for scientific mapping. Analyses can be used to determine which papers in a field of study should be prioritised. Although there are several ways to evaluate the significance of publications in a scientific topic (such as network metrics), their citation is the easiest and most precise approach to determining their impact [82].

#### 3.2.2. Co-Citation Analysis

A science mapping technique called co-citation analysis [83] takes the assumption that books that are frequently cited together have similar themes. The authors claim that by using analysis, it is possible to discover a research field’s conceptual framework as well as any underlying themes. When two publications are cited together in the references list of another article, they are connected by a co-citation network. Co-citation analysis can help business researchers find the most significant publications and learn about subject clusters [83]. These subject groups are built on the foundation of the mentioned papers.

#### 3.2.3. Bibliographic Coupling

A technique for science mapping called bibliographic coupling is predicated on the notion that two publications with comparable references also have comparable content [83]. The analysis focuses on grouping articles into themes based on common references, and it functions best within a certain historical period.

#### 3.2.4. Co-Word Analysis

Notable terms can also be gathered for the analysis from “article titles,” “abstracts,” and “full texts” when author keywords are not accessible. In co-word analysis, author keywords are typically employed to generate the words [83].

#### 3.2.5. Co-Authorship Analysis

The relationships between researchers are investigated via co-authorship [84] analysis. Understanding how academics interact is crucial since co-authorship is a formalised form of academic collaboration (including linked author qualities such affiliated institutions and nations).

### 3.3. Dataset

The table gives a general overview of the bibliometric data frame of the 214 publications that were chosen after conducting a thorough search on the Scopus database, and it divides the entire extracted information into the following sections, including main information, document contents, authors, authors’ collaborations, and document types. These chosen studies have 134 journal and book sources totalling 4127 references. The average number of articles cited per document may be 9.916 with a 50.08 percent yearly growth rate. Additionally, these papers have 503 unique author keywords from 678 writers in addition to a total of 1114 keywords. The data collection includes 4.36 co-authors and 14.02% international co-authorship, with 15 authors working alone. Articles, book chapters, conference papers, conference reviews, data papers, and reviews are among the several categories into which the documents are separated. The number of all documents is shown in the Table 1 below.

### 3.4. Publication and Citation Structure

**Figure 13:**
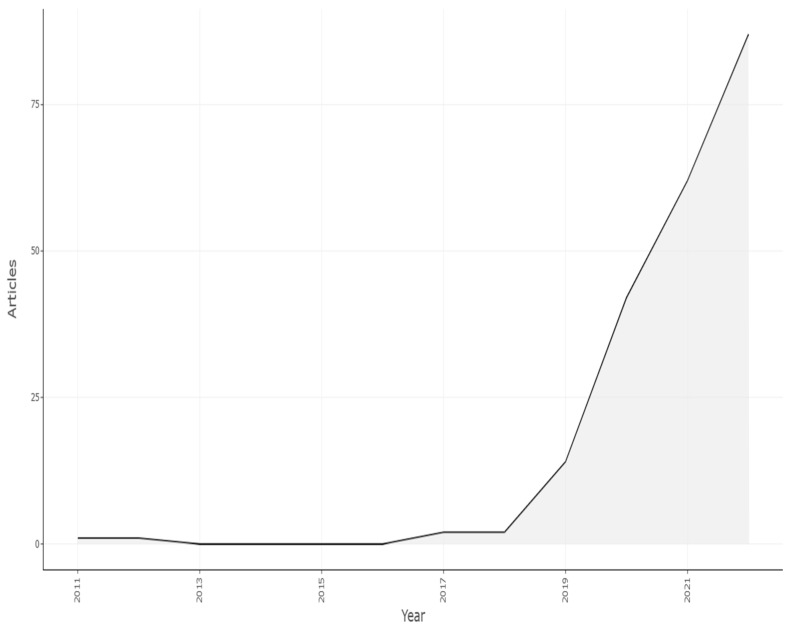
Scientific Article Production by Year. Data access information source: http://www.scopus.com (accessed on 14 September 2022).

In order to conduct this study, records pertaining to apple leaves over the past 11 years, from 2011 to 2022, have been acquired. The accompanying graph, Figure 13, displays annual trends in the publication of apple disease.

This information makes it clear that the research field is still developing and that it made more contributions after 2018 in increasing order, which is shown in Graph 1. Although the total number of publications in 2022 is not yet complete (the search was carried out during the year), we can clearly notice an upward trend since 2018. As a result, this subject is not only receiving more attention but is also relatively new.

The table displayed citation years, average total citations per article, average total citations per year, and the number of papers produced annually. In terms of citation patterns, there is no discernible pattern other than the fact that older publications are mentioned more frequently. The document citation rate of all studies will be described in Table 2 and Figure 13.

The Table 2 displayed citation years, average total citations per article, and the number of articles published annually. In terms of citation structure, there is no clear trend, aside from the obvious fact that the older the publication, the more it is cited.

### 3.5. Three Field Plot

**Figure 14:**
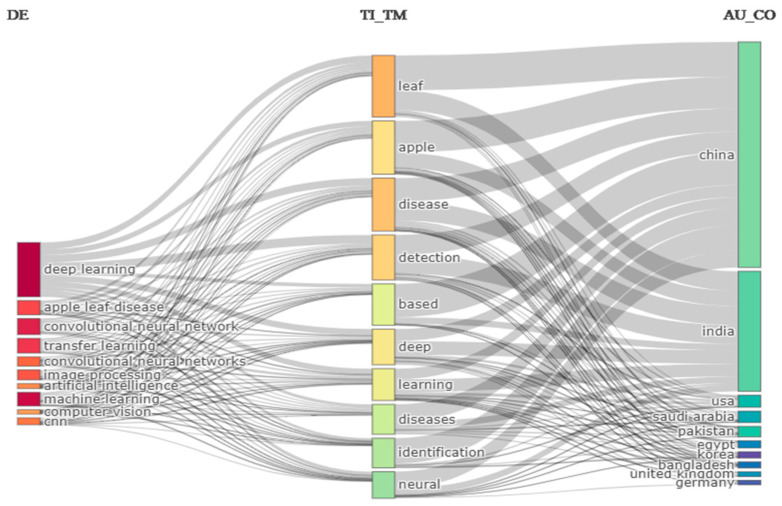
Three Field Plot (Sankey Diagram) of Country, Keywords and References of cited references of publication for the ten most researched topics.

A three-field plot (Sankey diagram) [85] of country, keyword, and year of publication of the cited references was developed to illustrate the proportion of study topics for each country and the recentness of the papers that they cited. Apple leaf disease detection is the primary area of interest for researchers in China and India, as illustrated in Figure 14. The researcher mainly focused on deep learning and machine learning techniques for the detection of disease. The majority of studies that addressed consent have been released by the USA, Saudi Arabia, and Pakistan. Despite their rarity, Egypt, Korea, the UK, and Germany also play important roles in their collaboration.

### 3.6. Relevant Sources

**Figure 15:**
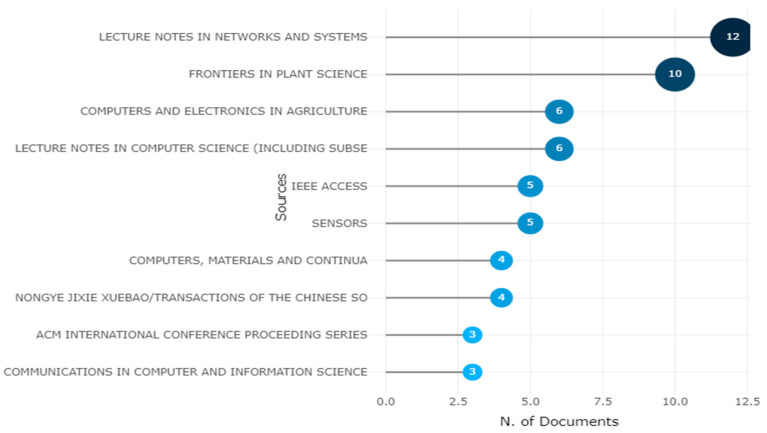
Ten Topmost Relevant Publication Sources of Apple Leaves Disease. Data access information source: http://www.scopus.com (accessed on 14 September 2022).

The below 15 figures will illustrate the most relevant sources of research published on different platforms. The “Lecture Notes in Networks and Systems” journal is the most prominent source for obtaining the 12 articles for this study, followed by “Frontiers in Plant Science” and “Communication in Computer and Information Science.” The graphic depicts the ten most popular high impact factor journals, with citations serving as a barometer of a journal’s reputation shown in Figure 15.

### 3.7. Local Cited Sources

**Figure 16:**
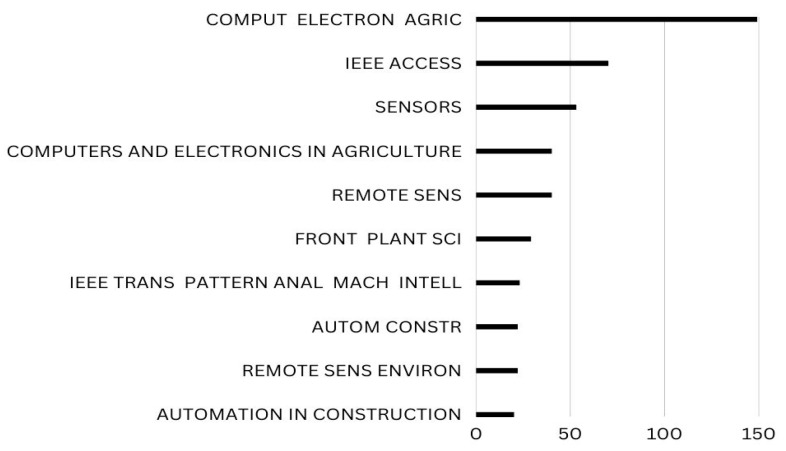
Top Ten Local Cited Sources in the field of Apple Leaves Disease. Data access information source: http://www.scopus.com (accessed on 14 September 2022).

Local citations are references which a document or author receives from another document already incorporated into the current study. Global citations, on the other hand, refer to all the citations (TC) that a piece of writing has received from sources indexed in a bibliographic database (WoS, Scopus, etc.). With maximum local citations each, COMPUT ELECTRON AGRIC and IEEE ACCESS are ranked top in Figure 16.

### 3.8. Source Dynamics

LOWESS [86] (locally weighted estimated scatter plot smoothing), which represents the source growth of the top six journals, is used to illustrate the number of publications over time. According to this figure, research on apple leave disease just started in 2017 and afterwards. This could lead to the creation of a field of multidisciplinary study. The large number of journals that cover the research topic suggests that it has a wider range of study issues and is multidisciplinary. Based on annual occurrences, the peak time for this study will gradually increase between 2020 and 2022, as shown in Table 3.

### 3.9. Most Relevant Affiliation

**Figure 17:**
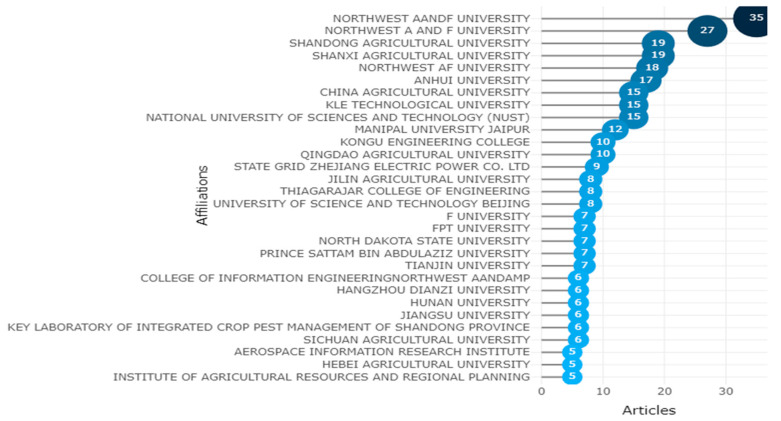
Affiliation Statistics of Apple Leaves Diseases. Data access information source: http://www.scopus.com (accessed on 14 September 2022).

Accordingly, the citation is a tool for connecting ideas and arguments as well as documents. As a result, the citation analysis can be used to gauge how different entities—such as countries, universities, research institutes, or journals—have an impact on the research process and to monitor how their performance has changed over time. The institutions with the highest rates of citation for academic articles authored by alumni are shown in the graph below. Northwest A&F University in China will receive the most article affiliations, while Zayed University in Dubai will receive the fewest, as shown in Figure 17.

### 3.10. Country’s Production Overtime

**Figure 18:**
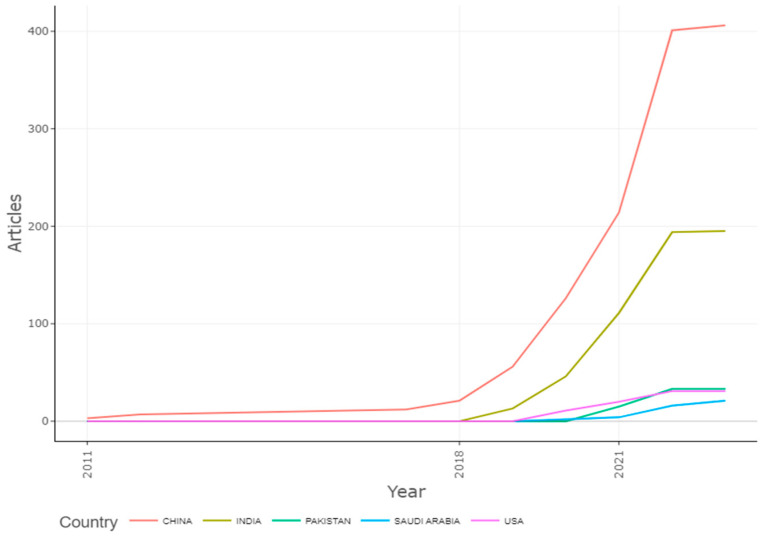
Country’s Production Overtime of Apple Leaves Disease. Data access information source: http://www.scopus.com (accessed on 14 September 2022).

Figure 18 shows that the detection of apple leaf diseases has shifted significantly in China. Numerous techniques for the detection of diseases in apples will be applicable, and this study will further work in India, so that is why India reserved the second post for this investigation after 2018 and was followed by Pakistan, Saudi Arabia, and the USA.

### 3.11. Documents

**Figure 19:**
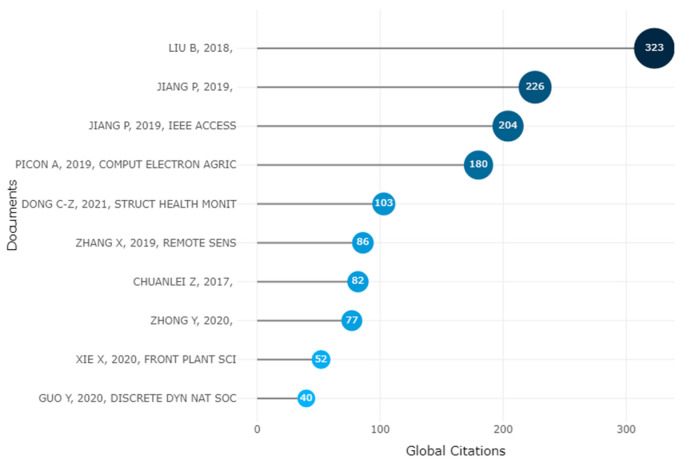
Ten Topmost Global Citations of Apple Leaves Disease [25,87,88,89,90]. Data access information source: http://www.scopus.com (accessed on 14 September 2022).

The number of citations received by a document from all documents in the database is calculated using global citations, which are used to identify highly cited publications. The number of citations a document receives from other documents in the collection under consideration is calculated using local citations. Figure 19 displays the papers that Scopus determined to be highly cited.

### 3.12. WordCloud

Word clouds have become a popular and appealing way to visualise text. They are used to summarise information by leaving out all but the most common terms in a variety of scenarios.

**Figure 20:**
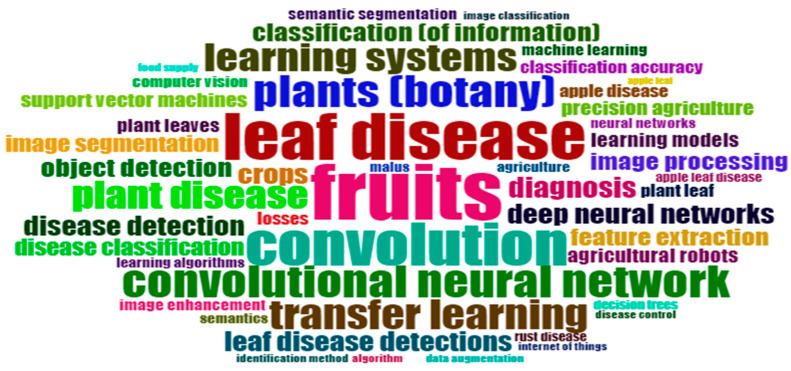
WordCloud of Apple Leaves Disease, DL/ML Keywords (Source: Author elaboration using Biblioshiny).

To accomplish this, static text summaries are frequently employed [91]. Word clouds produced for a body of text can provide a jumping-off point for more in-depth investigation. The above Figure 20 displays a word cloud created utilising Scopus statistics and buzzwords.

### 3.13. TreeMap

**Figure 21:**
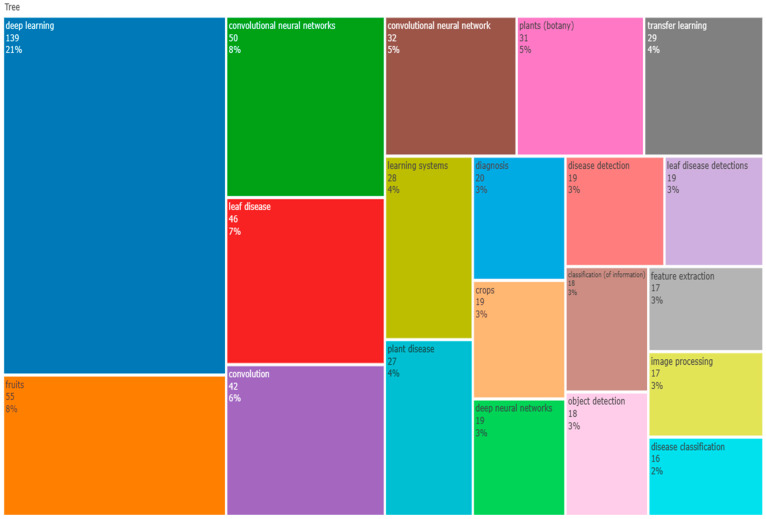
World TreeMap (Source: Author elaboration using Biblioshiny).

A visualisation called a tree map [92] was created expressly to make it easier to explore data that are organised hierarchically and in trees. The research trend, debate gaps, and related fields that would be of interest as research areas could all be identified using this data. The top 50 terms that appear most frequently in the articles are displayed in a word tree map below Figure 21. The tree map in the image represents the intersection of leaf disease, deep learning, convolutional neural networks, apple disease, machine learning, and diagnosis keywords.

## 4. Scientific Mapping

Science mapping aims to create bibliometric maps that show how particular disciplines, scientific subfields, or research sectors are conceptually, intellectually, and socially organised. For science mapping analysis, a wide range of methods and software packages have been suggested. An important area of study in the subject of bibliometrics is science mapping [93], often known as bibliometric mapping. Within the dynamic structure of scientific knowledge, it searches for instances of conceptual connections. Science mapping aims to depict the dynamic and structural aspects of scientific study.

### 4.1. Conceptual Approach

Scholars frequently utilise conceptual structures to comprehend the topics covered by scholars in order to determine which topics are more recent and relevant. Conceptual structure offers perception into the topography of a scientific topic using correspondence analysis (CA) [94], multiple correspondence analysis (MCA) [95], metric multidimensional scaling (MDS) [96], and clustering of a bipartite network of phrases collected from keywords, titles, or abstracts. The conceptual structure is further divided into two approaches: network and factorial.

Numerous network types are discovered through bibliometric analysis, particularly citation networks (in which links serve as substitutes for bibliographic citations) and cooperation networks (in which links correspond to article co-authorships). The network plot function uses VOSviewer or R scripts to visualise a network created by Biblio Network. To capture key facets of the underlying research system, the function of bibliometric units such as scholars and journals is specifically explored.

#### 4.1.1. Keyword Co-Occurrence Network

**Figure 22:**
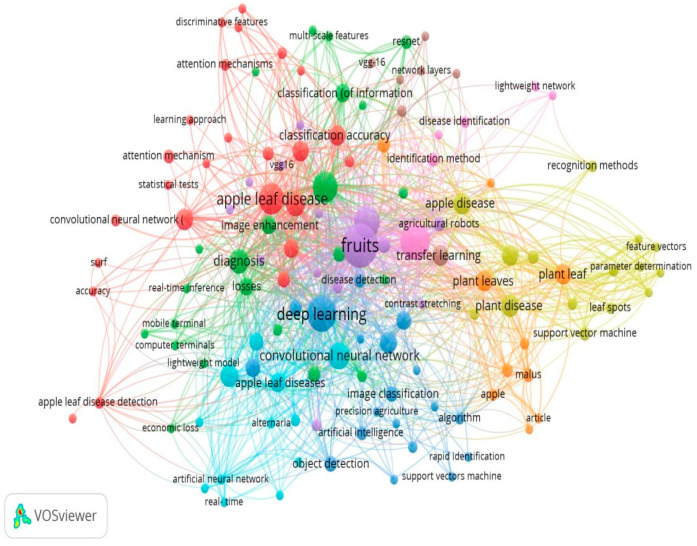
Structure of Keyword Wise Co-occurrence Network (Source: https://www.vosviewer.com (accessed on 14 September 2022)).

To examine the research on apple leaf disease and deep learning, the visualisation tool VOSviewer employed the keyword co-occurrence clumping view, keywords co-occurrence sequencing view, and keyword co-occurrence clustering frequency view. The VOSviewer software was used to create a keyword co-occurrence clustering view in the fields of deep learning and apple leaf disease. Out of the 519 terms with a frequency of less than 30, 131 were selected for co-occurrence analysis, as shown in Figure 22. The top 131 keywords will be chosen for each, and the overall strength of their co-occurrence with other keywords will be assessed.

Cluster analysis of the built-in clusters in the network visualisation map is also taken care of to display the study patterns. Pigments are used to symbolise each cluster. Nine clusters are shown in the above figure, the first of which is represented by a cluster of red bubbles and contains 25 things such as “accuracy, apple leaf, apple leaf disease, apple leaf disease detection, attention mechanism, classification accuracy, computer vision and image segmentations, etc.” A total of 23 items, including “apple leaves, attentiveness, categorization, computer terminals, crops, detection performance, diagnosis, disease control, and image enhancement,” are included in Cluster 2, which is represented by a green bubble. Eighteen things, including “deep learning, contrast, image classification, precision agriculture, artificial intelligence, object detection, fast identification, support vector machine, and algorithm,” are included in Cluster 3, which is represented by a blue bubble. Nine elements, including “identification technique, apple disease, apple leaves, apple leaf, svm, malus, apple, and article,” are included in Cluster 4, which is indicated by the hue of the bubbles.

On the basis of examination of the co-occurring keywords in the title and abstract, the following are some insights for future research.

- How can various artificial intelligence applications be used effectively in apple crop disease detection techniques?
- How computer vision is more reliable for processing of data?
- Which transfer learning is use for training of data of disease detection?
- How to evaluate performances matrices such as precision for different classes of apple leaf diseases?
- Which timeline is applicable in research for finding the best solution from all over the world intended for detection of ailments in apple leaves?
- What are the various datasets available for apple leaf disease detection in DL?
- What is the role of a deep learning model for automatic apple leaf disease detection?
- How does black spot affect apple production?
- What are the various manual and automatic detection techniques which can be used to tackle this problem?
- Which DL model shows significant efficiency in apple leaf detection?
- Why and how do leaf diseases affect agriculture yield?
- What are the various kinds of apple leaf diseases?
- What are practices for the farmers to identify apple leaf diseases?
- How do plant disease detection techniques affect the variety of crops?

#### 4.1.2. Thematic Map

**Figure 23:**
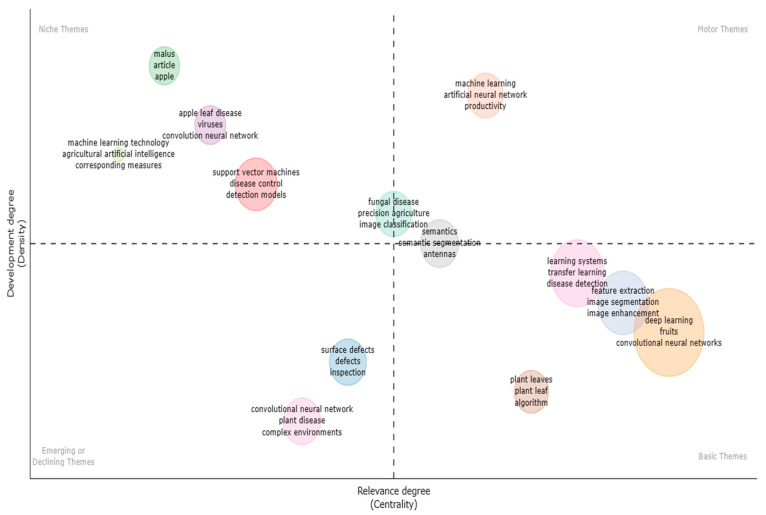
Thematic Map (Source: Author elaboration using Biblioshiny).

Thematic maps [97], similar to other maps, are made up of standard core parts such as algebra, geography, socioeconomics, and auxiliary and additional features. Thematic maps are distinguished by their diversity in content, purpose, graphics, and scale. The content of thematic maps combines topographic background and thematic content, in contrast to that of topographic maps, whose content is made up of planimetry and hypsography. The below Figure 23 is divided into four quadrants, with themes such as “basic,” “motor,” “niche,” and “developing” in each quadrant. Each theme comprises information that is supported by bubbles of various colours. Beginning with the Basic theme, there were algorithms for apple leaves, feature extraction, illness detection, image segmentation, and transfer learning. Second, the motor theme supports productivity, artificial neural networks, and semantics. Thirdly, the niche theme elaborated on viruses, support vector machines, machine learning, and apple leaf disease. Finally, the emerging or declining theme reflected scrutiny, complex environments, surface flaws, and plant diseases.

#### 4.1.3. Factorial Analysis

**Figure 24:**
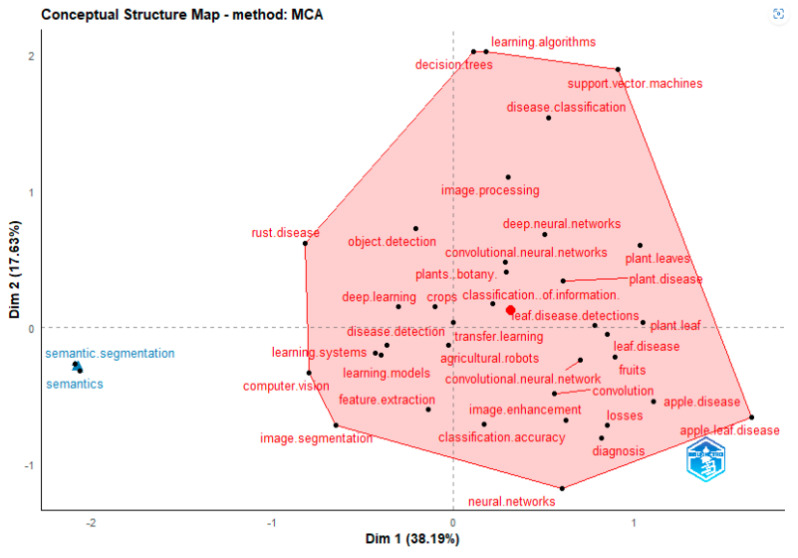
Factorial Analysis (Source: Author elaboration using Biblioshiny).

Bibliometrix may consider keywords as well as terms from article titles and abstracts. Utilising multiple correspondence analysis, correspondence analysis (CA), and network analysis, this is accomplished (MCA). The conceptual framework is shown as a two-dimensional layout Figure 24 by CA and MCA.

Deep learning, machine learning, learning algorithms, food supply, support vector machines, disease classification, image pro−cessing, crops, object detection, apple leaves, agriculture, feature extraction, apple leaves disease, losses, disease control are all covered by Cluster 1 (red). These keywords are associated with deep learning and machine learning techniques for artificial intelli−gence and the detection of apple leaves diseases. Cluster 2 (blue) introduces image segmentation concept such as semantic seg−mentation and semantics.

## 5. Intellectual Structure

By analysing the interactions between writers and nations, the intellectual structure develops deductions about how various authors influence the scientific community. The research fraternity collaborates, as shown by an analysis of citations and co-citations, as well as by the affiliations of these groups with various institutions.

### 5.1. Bibliographic Coupling with Countries

Bibliographic coupling [98] is the act of two works citing a third work in their respective bibliographies. You can make a bibliographic coupling map in VOSviewer by using Documents, Sources, Authors, Organisations, and Countries. Through a variety of studies and research articles, Kessler introduced bibliographic coupling to the scientific community in the 1960s. It was mainly promoted as a framework for organising technical and scientific material to hasten knowledge transfer and document retrieval.

### 5.2. Document X Citations

**Figure 25:**
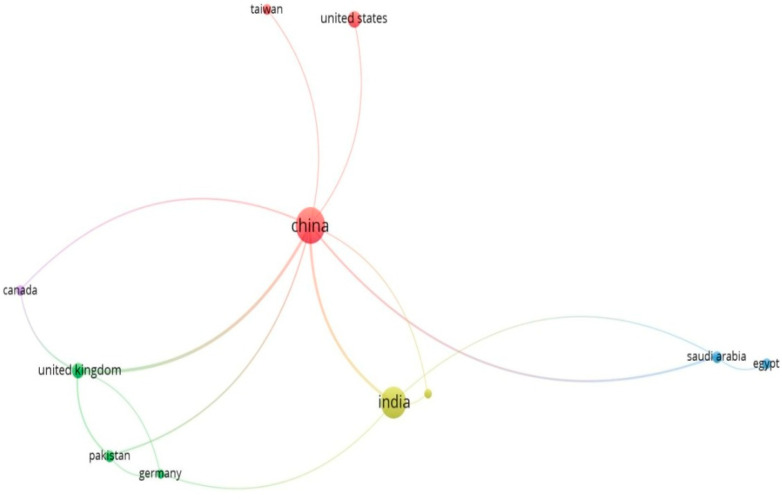
Country Wise Document X Citation Network (Source: https://www.vosviewer.com (accessed on 14 September 2022)).

The criteria, which were set at a minimum of three documents and citations per nation, are met by 12 of the 44 countries. The total strength of each country’s bibliographic coupling relationships to the other nations will be determined for each of the 12 countries which are shown in given Table 4 and Figure 25.

China had the most articles written, pursued by India, Pakistan, Canada, Taiwan and South Korea. India, China, Germany, and the United State received the most citations. The cluster analysis revealed four groups of countries.

### 5.3. Co-Citation Analysis

**Figure 26:**
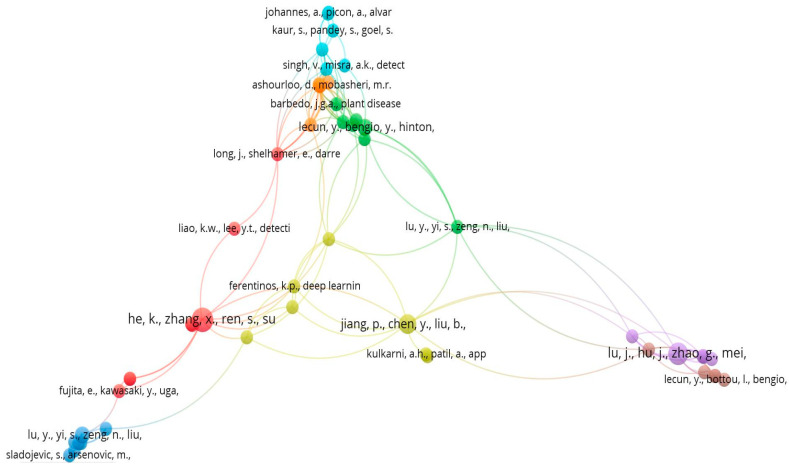
Reference Wise Co-citation Analysis Network (Source: https://www.vosviewer.com (accessed on 14 September 2022)).

According to this analysis out of the total cited reference such as 4117, a minimum number of citations of cited reference will be selected, and after this selection, 68 meet the threshold value which is shown in below Figure 26. Co-citations of papers are used to conduct searches on related documents. When collection administrators build core journal lists, select journals, and evaluate collections, journal co-citation is crucial. Author co-citation analysis has been used to analyse the intellectual structure of research domain.

#### 5.3.1. Co-Citation Source

**Figure 27:**
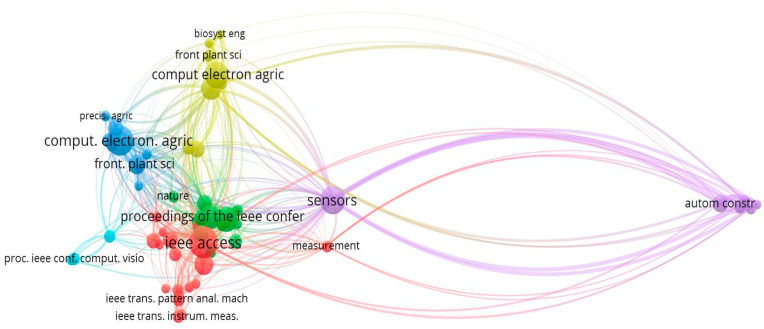
Journal Wise Co-citation Sources Network (Source: https://www.vosviewer.com (accessed on 14 September 2022)).

According to this analysis out of the total cited reference such as 2256, the minimum number of citations of a cited reference that will be selected is 7, and after this selection 60 meet the threshold value which is shown in below Figure 27.

#### 5.3.2. Co-Citation Author

**Figure 28:**
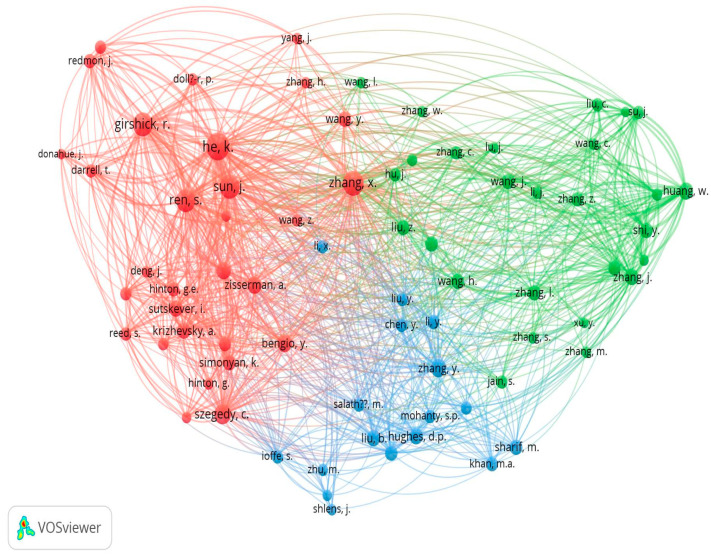
Author Wise Co-citation Network (Source: https://www.vosviewer.com (accessed on 14 September 2022)).

According to this analysis out of the total cited reference such as 6736, the minimum number of citations of a cited reference that will be selected is 15, and after this selection, 74 meet the threshold value which is shown in below Figure 28.

## 6. Social Structure

The social system in the subject of apple disease detection scientific research demonstrates how writers, institutions, or nations relate to one another [99].

### 6.1. Collaboration Network

There has been an increase in interest in research collaboration during the past few decades. Some evidence for increased scientific collaboration can be seen in the observed rise in co-authorship. Researchers collaborate for a variety of reasons, including the need to address complicated research issues, the need for learning and productivity in the field, the need to lower research costs, and the need for intellectual company. Scholars have started working together more regularly as research techniques and theories have grown more complicated. Collaboration amongst academics can enhance research; for instance, multiple viewpoints might offer more depth and clarity. Collaboration among authors can inevitably lead to collaboration between institutions and countries. The same idea is shown in the diagram below.

#### 6.1.1. Institute-Wise Collaboration

The word “collaboration” implies that a group of independent researchers are working together to accomplish a common objective. Collaboration between institutions enables researchers to interact with peers who are experts in a particular field of study, gain access to necessary resources such as databases, staff, equipment, and study populations, and gain credibility from the well-known names of eminent researchers, departments, and institutions [100].

**Figure 29:**
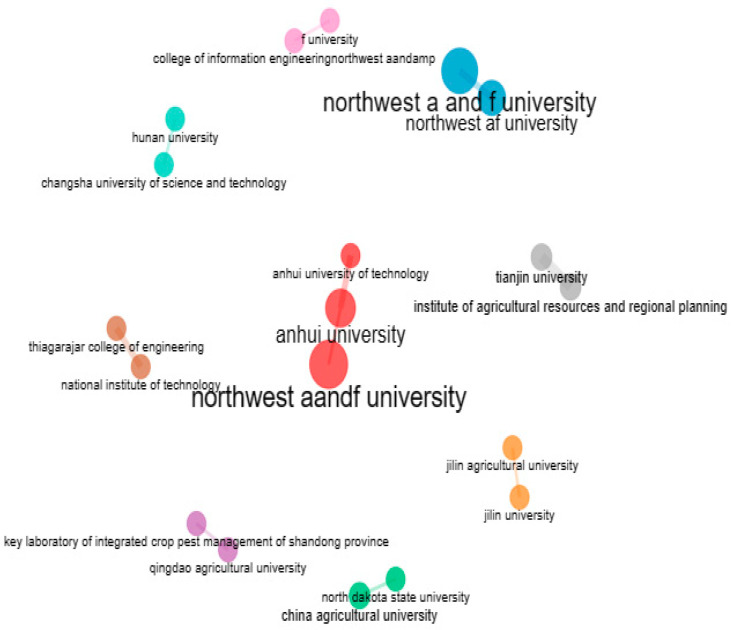
Structure of Co-authorship relationships between Institute (Source: Author elaboration using Biblioshiny).

When addressing research questions, the practice of collaborating with teams from various institutions can provide new perspectives. Collaboration between institutions offers a way to grow one’s network of research contacts, which could eventually result in additional collaboration. Figure 29 depicts a network of affiliations among institutes that have co-authored articles with them. Author clusters with strong network connections are represented by the colours. It is clear that there is collaboration between Northwest A and F Universities, Anhui University, and Changsha University because they are all included in the same cluster (Blue).

#### 6.1.2. Country-Wise Collaboration

**Figure 30:**
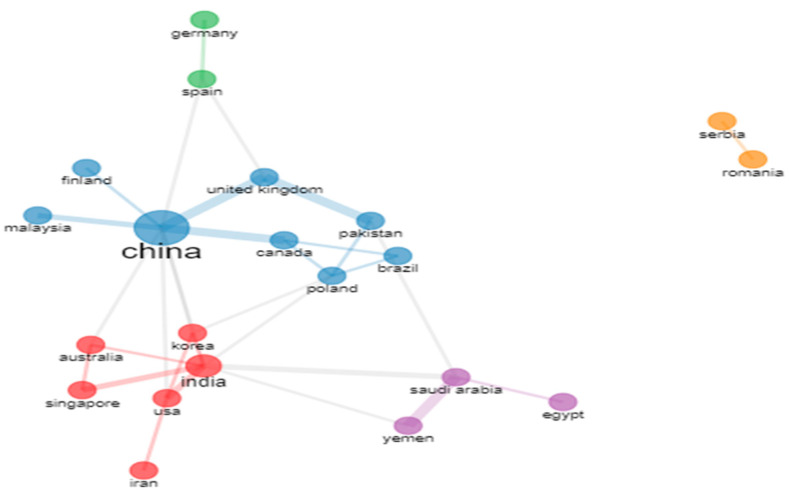
Structure of Co-authorship relationships between Countries (Source: Author elaboration using Biblioshiny).

The below Figure 30 displays the network of co-authorship connections between countries that have papers published with them. Coloured clusters indicate country clusters with significant network connections.

### 6.2. Collaboration WorldMap

**Figure 31:**
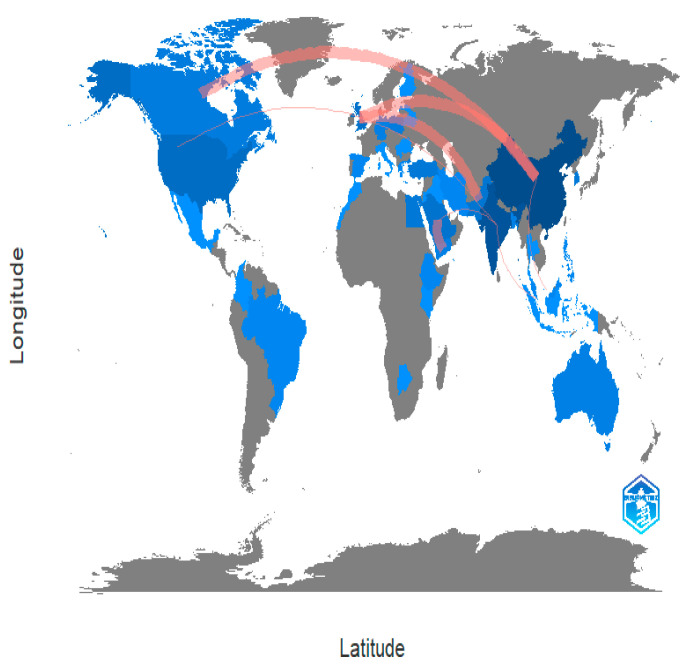
Spatial Distribution of Total Publications by Country (Source: Author elaboration using Biblioshiny).

Academic research is collaborating more internationally. It is better for their work when researchers are corresponding with their colleagues all around the world, whether it is to find specialised tools, generate fresh concepts, or find new funding sources. International collaboration in academic research is becoming more common. Researchers are contacting their peers all over the world, which is beneficial to their work, whether it is to acquire specialised equipment, develop new ideas, or gain access to new funding sources [101,102]. In the time of a pandemic, scientists and researchers across borders and disciplines have come together in inspiring ways. In Figure 31, it can be seen how the world is working together to combat apple leaves.

## 7. Conclusions

This article highlights the development of apple leaf disease detection techniques over the years from 2011 to 2023, utilising deep learning and machine learning techniques. Additionally, it offers a wide-ranging investigation of the theoretical, scholarly, and communal foundations of the research issue. The study’s main contribution is the organisation of the field’s fragmented literature and the identification of key sources, authors, and documents. Due to their adaptability and user-friendliness, the bibliometrics tools Bibliometrix R-package and VOSviewer are employed. These data imply that research on apple illnesses only started in 2018 and later. Since 2018, the volume of publications in the Lecture Notes in Network and Systems has grown quickly. This could lead to the creation of a field of multidisciplinary study. Local citations are references that an author or document receives from another work that has already been incorporated into the current investigation. Global citations, on the other hand, refer to all the citations (TC) that a piece of writing has received from sources indexed in a bibliographic database (WoS, Scopus, etc.). In this study, COMPUTER ELECTRON AGRIC, which has 73 local citations, is ranked first. IEEE Access is in second place with 70 local citations. As a result, the ambition of this exploration is to extend a road map for academics and researchers to understand the body of existing information. Additionally, users can utilise bibliometric analysis to look at publication patterns in terms of authors, citations, sources, nations, high-impact papers by well-known authors, international collaboration, and theme development.

## 8. Limitations and Future Directions

For this analysis, only papers listed in Scopus were taken into account. The current research investigation may not have taken into account all scientific articles from some of the other research databases, such as Web of Science and Google Scholar. The Scopus database search terms were chosen by the authors. Depending on the researcher’s perspective, the set of keywords used for analysis might be revised, altered, and rearranged. The articles from 2011 to 2023 are taken into account for the outcomes analysis. For this research study, only documents written in English are taken into consideration. The secondary sources listed in Scopus are not included in the research paper. For additional research purposes, those documents can also be used. The hottest subjects may be investigated in future studies. Several academics have become interested in using the Explainable AI (XAI) technique to offer explanations for forecasts.

## Figures and Tables

**Table 1:** Detailed description of the chosen dataset along with the study’s results.

Description	Results
MAIN INFORMATION ABOUT DATA	
Timespan	2011:2022
Sources (Journals, Books, etc)	134
Documents	214
Annual Growth Rate %	50.08
Document Average Age	2.08
Average citations per doc	9.916
References	4127
DOCUMENT CONTENTS	
Keywords Plus (ID)	1114
Author’s Keywords (DE)	503
AUTHORS	
Authors	678
Authors of single-authored docs	5
AUTHORS COLLABORATION	
Single-authored docs	15
Co-Authors per Doc	4.32
International co-authorships %	14.02
DOCUMENT TYPES	
Article	67
Book chapter	5
conference paper	55
conference review	8
data paper	1
Review	3

Data access information source: http://www.scopus.com (accessed on 14 September 2022).

**Table 2:** Annual Citation Structure per year.

Year	N	MeanTCperArt	MeanTCperYear	CitableYears
2011	1	1.00	0.09	11
2012	1	27.00	2.70	10
2013	0	0.00	0.00	0
2014	0	0.00	0.00	0
2015	0	0.00	0.00	0
2016	0	0.00	0.00	0
2017	2	41.00	8.20	5
2018	2	172.50	43.13	4
2019	14	54.36	18.12	3
2020	42	10.48	5.24	2
2021	62	5.63	5.63	1
2022	87	1.34		0

Data access information source: http://www.scopus.com (accessed on 14 September 2022).

**Table 3:** Annual Dynamic Occurrences of Articles Year Wise of Apple Leaves Disease.

Year	Lecture Notes in Networks and Systems	Frontiers in Plant Science	Computers and Electronics in Agriculture	Lecture Notes in Computer Science (Including Subseries Lecture Notes in Artificial Intelligence and Lecture Notes in Bioinformatics)	Sensors	Ieee Access
2011	0	0	0	0	0	0
2012	0	0	0	0	0	0
2013	0	0	0	0	0	0
2014	0	0	0	0	0	0
2015	0	0	0	0	0	0
2016	0	0	0	0	0	0
2017	0	0	0	1	0	0
2018	0	0	0	1	0	0
2019	0	0	1	2	2	0
2020	0	1	2	3	2	0
2021	5	5	3	5	4	3
2022	12	10	6	6	5	5

Data access information source: http://www.scopus.com (accessed on 14 September 2022).

**Table 4:** Structure of Connections in the literature Country Wise.

Country	Documents	Citations	Total Link Strength
China	44	516	23
India	33	140	9
United Kingdom	8	106	12
Pakistan	5	15	5
Saudi Arabia	5	24	5
Canada	4	6	4
Germany	3	189	3
South Korea	3	31	2
Egypt	4	2	1
Taiwan	4	27	1
United State	9	123	1
Australia	3	13	0

## Data Availability

Not Applicable.

## References

[1] Samajpati B.J., Degadwala S.D. (2015). A Survey on Apple Fruit Diseases Detection and Classification. Int. J. Comput. Appl..

[2] Khirade S.D., Patil A. Plant disease detection using image processing. Proceedings of the 2015 International Conference on Computing Communication Control and Automation.

[3] Rao A., Kulkarni S. (2020). A Hybrid Approach for Plant Leaf Disease Detection and Classification Using Digital Image Processing Methods. Int. J. Electr. Eng. Educ..

[4] Phadikar S., Sil J., Das A.K. (2013). Rice diseases classification using feature selection and rule generation techniques. Comput. Electron. Agric..

[5] Rastogi A., Arora R., Sharma S. Leaf disease detection and grading using computer vision technology & fuzzy logic. Proceedings of the 2015 2nd International Conference on Signal Processing and Integrated Networks (SPIN).

[6] Singh R., Gehlot A., Prajapat M.K., Singh B. (2021). Artificial Intelligence in Agriculture.

[7] Li L., Zhang S., Wang B. (2021). Apple Leaf Disease Identification with a Small and Imbalanced Dataset Based on Lightweight Convolutional Networks. Sensors.

[8] Di Franco G., Santurro M. (2020). Machine learning, artificial neural networks and social research. Qual. Quant..

[9] Abdullah D.M., Abdulazeez A.M. (2021). Machine Learning Applications based on SVM Classification A Review. Qubahan Acad. J..

[10] Mitra M. (2019). K-Means Clustering in Machine Learning—A Review. Peer Nest.

[11] Catherine A.O. (2013). Fuzzy C-Means Clustering Model for Identification of Students ’ Learning Preferences in Online Environment. Int. J. Comput. Appl. Inf. Technol..

[12] Yang L., Wu X., Zhao D., Li H., Zhai J. An improved Prewitt algorithm for edge detection based on noised image. Proceedings of the 2011 4th International Congress on Image and Signal Processing.

[13] Doinea M., Boja C. Machine Learning Techniques for Data Extraction and Classification in Computer Vision Software. Proceedings of the 13th International Conference on INFORMATICS in ECONOMY (IE 2014).

[14] Guseva A.I., Kuznetsov I.A. The Use of Entropy Measure for Higher Quality Machine Learning Algorithms in Text Data Processing. Proceedings of the 2017 5th International Conference on Future Internet of Things and Cloud Workshops (FiCloudW).

[15] Chai T., Draxler R.R. (2014). Root mean square error (RMSE) or mean absolute error (MAE)?–Arguments against avoiding RMSE in the literature. Geosci. Model Dev..

[16] Balanda K.P., MacGillivray H.L. (1988). Kurtosis: A Critical Review. Am. Stat..

[17] Oravec M. Feature extraction and classification by machine learning methods for biometric recognition of face and iris. Proceedings of the ELMAR-2014.

[18] Dubey S.R., Jalal A.S. Detection and classification of apple fruit diseases using complete local binary patterns. Proceedings of the 2012 Third International Conference on Computer and Communication Technology.

[19] Shuaibu M., Lee W.S., Schueller J., Gader P., Hong Y.K., Kim S. (2018). Unsupervised hyperspectral band selection for apple Marssonina blotch detection. Comput. Electron. Agric..

[20] Abbasi P.A., Ali S., Braun G., Bevis E., Fillmore S. (2019). Reducing apple scab and frogeye or black rot infections with salicylic acid or its analogue on field-established apple trees. Can. J. Plant Pathol..

[21] Sherwani A., Mukhtar M. (2016). Insect Pests of Apple and Their Management Fishery biology View Project Toxicology View Project. https://www.researchgate.net/publication/290053683.

[22] Singh S., Gupta S., Tanta A., Gupta R. (2021). Extraction of Multiple Diseases in Apple Leaf Using Machine Learning. Int. J. Image Graph..

[23] Chandel A.K., Khot L.R., Sallato B.C. (2021). Apple powdery mildew infestation detection and mapping using high-resolution visible and multispectral aerial imaging technique. Sci. Hortic..

[24] Kodors S., Lacis G., Sokolova O., Zhukovs V., Apeinans I., Bartulsons T. (2021). Apple scab detection using CNN and transfer learning. Agron. Res..

[25] Jiang P., Chen Y., Liu B., He D., Liang C. (2019). Real-Time Detection of Apple Leaf Diseases Using Deep Learning Approach Based on Improved Convolutional Neural Networks. IEEE Access.

[26] Fang T., Chen P., Zhang J., Wang B. (2019). Identification of Apple Leaf Diseases Based on Convolutional Neural Network. Lecture Notes in Computer Science (Including Subseries Lecture Notes in Artificial Intelligence and Lecture Notes in Bioinformatics).

[27] Alsayed A., Alsabei A., Arif M. (2021). Classification of Apple Tree Leaves Diseases using Deep Learning Methods. Int. J. Comput. Sci. Netw. Secur..

[28] Chao X., Sun G., Zhao H., Li M., He D. (2020). Identification of Apple Tree Leaf Diseases Based on Deep Learning Models. Symmetry.

[29] Bonkra A., Noonia A., Kaur A. (2022). Apple Leaf Diseases Detection System: A Review of the Different Segmentation and Deep Learning Methods. Artif. Intell. Data Sci..

[30] Fu K., Mui J. (1981). A survey on image segmentation. Pattern Recognit..

[31] Shrivastava P. (2013). A Survey of Image Classification Based Techniques. Int. J. Eng. Res. Technol..

[32] Khalid S., Khalil T., Nasreen S. A survey of feature selection and feature extraction techniques in machine learning. Proceedings of the Science and Information Conference (SAI).

[33] Bonkra A., Dhiman P. IoT Security Challenges in Cloud Environment. Proceedings of the 2021 2nd International Conference on Computational Methods in Science & Technology (ICCMST).

[34] Rani R., Khurana M., Kumar A., Kumar N. (2022). Big data dimensionality reduction techniques in IoT: Review, applications and open research challenges. Clust. Comput..

[35] Rani R., Khurana M., Sharma D., Moudgil A. Comparative Study on various Storage Optimization Techniques in IoT-Cloud Ecosystem. Proceedings of the 2021 International Conference on Advance Computing and Innovative Technologies in Engineering (ICACITE).

[36] MacHardy E.W. (2000). Current status of IPM in apple orchards. Crop. Prot..

[37] https://www.apsnet.org/publications/plantdisease/backissues/Documents/1986Articles/PlantDisease70n05_480.pdf.

[38] Sholberg P., O’Gorman D., Bedford K., Lévesque C.A. (2005). Development of a DNA Macroarray for Detection and Monitoring of Economically Important Apple Diseases. Plant Dis..

[39] Chand-Goyal T., Spotts R.A. (1997). Biological Control of Postharvest Diseases of Apple and Pear under Semi-commercial and Commercial Conditions Using Three Saprophytic Yeasts. Biol. Control.

[40] Kotsiantis S., Zaharakis I., Pintelas P. (2007). Supervised machine learning: A review of classification techniques. Emerg. Artif. Intell. Appl. Comput. Eng..

[41] Panigrahi A., Chen Y., Kuo C.-C.J. (2018). Analysis on gradient propagation in batch normalized residual networks. arXiv.

[42] Seber G.A., Lee A. (2012). Linear Regression Analysis.

[43] Scholkopf B., Smola A. (2001). Learning with Kernels: Support Vector Machines, Regularization, Optimization, and Beyond.

[44] Jain A.K. (2009). Data clustering: 50 years beyond K-means. Pattern Recognit. Lett..

[45] Kotsiantis S., Kanellopoulos D. (2006). Association rules mining: A recent overview. GESTS Int. Trans. Comput. Sci. Eng..

[46] Mnih V., Badia A.P., Mirza M., Graves A., Lillicrap T.-T., Harley T., Silver D., Kavukcuoglu K. (2016). Asynchronous methods for deep reinforcement learning. arXiv.

[47] Albrektsson T., Johansson C. (2001). Osteoinduction, osteoconduction and osseointegration. Eur. Spine J..

[48] Kumar A.S. (2021). Image Segmentation and Object Recognition. J. Res. Proc..

[49] Umamaheswari D., Geetha S. (2019). Review on Image Segmentation Techniques Incorporated with Machine Learning in the Scrutinization of Leukemic Microscopic Stained Blood Smear Images.

[50] Kale R.S., Thorat S. (2021). Image Segmentation Techniques with Machine Learning. Int. J. Sci. Res. Comput. Sci. Eng. Inf. Technol..

[51] Smith K., Marinova D. (2005). Use of bibliometric modelling for policy making. Math. Comput. Simul..

[52] Donthu N., Kumar S., Mukherjee D., Pandey N., Lim W.M. (2021). How to conduct a bibliometric analysis: An overview and guidelines. J. Bus. Res..

[53] De Bakker F.G.A., Groenewegen P., den Hond F. (2005). A Bibliometric Analysis of 30 Years of Research and Theory on Corporate Social Responsibility and Corporate Social Performance. Bus. Soc..

[54] Ballew B.S. (2009). Elsevier’s Scopus^®^ Database. J. Electron. Resour. Med. Libr..

[55] Börner K., Huang W., Linnemeier M., Duhon R.J., Phillips P., Ma N., Zoss A.M., Guo H., Price M.A. (2010). Rete-netzwerk-red: Analyzing and visualizing scholarly networks using the Network Workbench Tool. Scientometrics.

[56] Sulaiman M.A. (2021). Introduction to Microsoft Excel. Introd. Data Sci..

[57] Yockey R.D. (2015). Introduction to SPSS. SPSS^®^ Demystified.

[58] Borgatti S., Everett M., Freeman L. (2002). Ucinet for Windows: Software for Social Network Analysis (Version 6.102).

[59] Batagelj V., Mrvar A. (1998). Pajek-Program for Large Network Analysis. Connections.

[60] Persson O., Danell R., Schneider J.W. (2009). How to use Bibexcel for various types of bibliometric analysis. Celebrating Scholarly Communication Studies: A Festschrift for Olle Persson at His 60th Birthday.

[61] Bailón-Moreno R., Jurado-Alameda E., Ruiz-Baños R., Courtial J.P. (2005). Analysis of the field of physical chemistry of surfactants with the Unified Scienctometric Model. Fit of relational and activity indicators. Scientometrics.

[62] Carvalho P., Hitzelberger P., Otjacques B., Bouali F., Venturini G. Information visualization for CSV open data files structure analysis. Proceedings of the 6th International Conference on Information Visualization Theory and Applications.

[63] Chen C. (2006). CiteSpace II: Detecting and visualizing emerging trends and transient patterns in scientific literature. J. Am. Soc. Inf. Sci. Technol..

[64] Chen C. (2004). Searching for intellectual turning points: Progressive knowledge domain visualization. Proc. Natl. Acad. Sci. USA.

[65] Wise J.A. (1999). The ecological approach to text visualization. J. Am. Soc. Inf. Sci..

[66] Peroni S., Osborne F., Di Iorio A., Nuzzolese A.G. (2017). Research Articles in Simplified HTML: A Web-first format for HTML-based scholarly articles. PeerJ Comput. Sci..

[67] Fernandez M., Amer-Yahia S. Techniques for storing XML. Proceedings of the 18th International Conference on Data Engineering.

[68] Hetzler E., Turner A. (2004). Analysis Experiences Using Information Visualization. IEEE Comput. Graph. Appl..

[69] Börner K., Biberstine J. (2011). Sci2: A Tool of Science of Science Research and Practice Tutorial. Please (1) Get a Name Tag, (2) Download the Sci2 Tool from http://sci2.cns.iu.edu and (3) Complete the Pre-Tutorial Questionnaire.

[70] Mahdi Q., Hashim R. (2020). Using R language to analyze and programming vital data by applying it to a human diseases. Int. J. Psychosoc. Rehabil..

[71] Burnham J.F. (2006). Scopus database: A review. Biomed. Digit. Libr..

[72] Williamson P.O., Minter C.I.J. (2019). Exploring PubMed as a reliable resource for scholarly communications services. J. Med. Libr. Assoc..

[73] Paramothayan N.S., Lasserson T.J., Wells A.U., Walters E.H. (2002). The cochrane database of systematic reviews. Ther. Res..

[74] Aria M., Cuccurullo C. (2017). bibliometrix: An R-tool for comprehensive science mapping analysis. J. Informetr..

[75] Van Eck N.J., Waltman L. (2010). Software survey: VOSviewer, a computer program for bibliometric mapping. Scientometrics.

[76] Francis B.R., bin Ahmad R., Abdullah S.M.B. (2021). A Bibliometric Analysis on Performance Appraisal. Int. J. Acad. Res. Bus. Soc. Sci..

[77] Leite L.R., Yagasaki C.A., Van Aken E., Martins R.A. Bibliometric analysis of literature on performance measurement systems and sustainability. Proceedings of the 2012 Industrial and Systems Engineering Research Conference.

[78] Chen C., Dubin R., Schultz T. (2014). Science Mapping. Encyclopedia of Information Science and Technology.

[79] Jarneving B. (2007). Bibliographic coupling and its application to research-front and other core documents. J. Informetr..

[80] Efron N., Brennan N.A., Nichols J.J. (2012). Citation Analysis of the Contact Lens Field. Optom. Vis. Sci..

[81] Osareh F. (1996). Bibliometrics, Citation Analysis and Co-Citation Analysis: A Review of Literature I. Libri.

[82] Surwase G., Sagar A., Kademani B.S., Bhanumurthy K. Co-citation Analysis: An Overview. Proceedings of the Creativity, Innovation and Discovery BOSLA National Conference.

[83] Khaldi H., Prado-Gascó V. (2021). Bibliometric maps and co-word analysis of the literature on international cooperation on migration. Qual. Quant..

[84] Ponomariov B., Boardman C. (2016). What is co-authorship?. Scientometrics.

[85] Fatehi F., Hassandoust F., Ko R.K., Akhlaghpour S. (2020). General data protection regulation (GDPR) in healthcare: Hot topics and research fronts. Stud. Health Technol. Inform..

[86] Pintus E., Sorbolini S., Albera A., Gaspa G., DiMauro C., Steri R., Marras G., Macciotta N.P.P. (2013). Use of locally weighted scatterplot smoothing (LOWESS) regression to study selection signatures in Piedmontese and Italian Brown cattle breeds. Anim. Genet..

[87] Liu B., Zhang Y., He D., Li Y. (2018). Identification of Apple Leaf Diseases Based on Deep Convolutional Neural Networks. Symmetry.

[88] Dong C.-Z., Catbas F.N. (2021). A review of computer vision–based structural health monitoring at local and global levels. Struct. Health Monit..

[89] Zhang X., Han L., Dong Y., Shi Y., Huang W., Han L., González-Moreno P., Ma H., Ye H., Sobeih T. (2019). A Deep Learning-Based Approach for Automated Yellow Rust Disease Detection from High-Resolution Hyperspectral UAV Images. Remote Sens..

[90] Zhong Y., Zhao M. (2020). Research on deep learning in apple leaf disease recognition. Comput. Electron. Agric..

[91] Heimerl F., Lohmann S., Lange S., Ertl T. (2014). Word Cloud Explorer: Text Analytics Based on Word Clouds. Proceedings of the 47th Hawaii International Conference on System Sciences.

[92] Scheibel W., Trapp M., Limberger D., Döllner J. A taxonomy of treemap visualization techniques. Proceedings of the 11th International Conference on Information Visualization Theory and Applications.

[93] Liu X. (2013). Full-Text Citation Analysis: A New Method to Enhance. J. Am. Soc. Inf. Sci. Technol..

[94] Kroonenberg P.M., Greenacre M.J. (2006). Correspondence Analysis. Encyclopedia of Statistical Sciences.

[95] Khangar N.V., Kamalja K.K. (2017). Multiple Correspondence Analysis and its applications. Electron. J. Appl. Stat. Anal..

[96] Briggs D. (2003). An Introduction to Multidimensional Measurement using Rasch models. J. Appl. Meas..

[97] Forrest D. (2015). Thematic Maps in Geography.

[98] Weinberg B.H. (1974). Bibliographic coupling: A review. Inf. Storage Retr..

[99] Lewis Y. (2021). Research collaborations bring big rewards: The world needs more. Nature.

[100] Pranckutė R. (2021). Web of Science (WoS) and Scopus: The Titans of Bibliographic Information in Today’s Academic World. Publications.

[101] Medhi B., Bansal S., Mahendiratta S., Kumar S., Sarma P., Prakash A. (2019). Collaborative research in modern era: Need and challenges. Indian J. Pharmacol..

[102] Geng Y., Zhu R., Maimaituerxun M. (2022). Bibliometric review of carbon neutrality with CiteSpace: Evolution, trends, and framework. Environ. Sci. Pollut. Res..

